# Dysregulation and antimetastatic function of *circLRIG1* modulated by *miR-214-3p/LRIG1* axis in bladder carcinoma

**DOI:** 10.1186/s13062-023-00446-x

**Published:** 2024-03-07

**Authors:** Shiliang Cheng, Chunguang Li, Lu Liu, Xinli Liu, Meng Li, Jinhua Zhuo, Jue Wang, Wen Zheng, Zhongmin Wang

**Affiliations:** 1 Department of Clinical Laboratory, Shandong Provincial Third Hospital, Cheeloo College of Medicine, Shandong University, Jinan Xingqi Medical Laboratory Co., Ltd., 12 Wuyingshan Middle Road, Jinan, 250000 Shandong China; 2 Department of Emergency, Shandong Provincial Third Hospital, Cheeloo College of Medicine, Shandong University, 12 Wuyingshan Middle Road, Jinan, 250000 Shandong China; 3 Department of Digestive Oncology, Cancer Hospital of Dalian University of Technology, Liaoning Cancer Hospital and Institute, 44 Xiaoheyan Road, ShenyangLiaoning, 110042 China; 4 Department of Clinical Laboratory, Shandong Provincial Third Hospital, Cheeloo College of Medicine, Shandong University, 12 Wuyingshan Middle Road, Jinan, 250000 Shandong China; 5 Department of Pharmacy, Shanghai Eastern Hepatobiliary Surgery Hospital, Navy Military Medical University, 225 Changhai Road, Shanghai, 200000 China

**Keywords:** Bladder carcinoma, *circLRIG1*, *miR-214-3p*, *LRIG1*

## Abstract

*CircLRIG1*, a newly discovered circRNA, has yet to have its potential function and biological processes reported. This study explored the role of *circLRIG1* in the development and progression of bladder carcinoma and its potential molecular mechanisms. Techniques such as qRT-PCR, Western blot, various cellular assays, and in vivo models were used to investigate mRNA and protein levels, cell behavior, molecular interactions, and tumor growth. The results showed that both *circLRIG1* and *LRIG1* were significantly reduced in bladder carcinoma tissues and cell lines. Low *circLRIG1* expression was associated with poor patient prognosis. Overexpressing *circLRIG1* inhibited bladder carcinoma cell growth, migration, and invasion, promoted apoptosis, and decreased tumor growth and metastasis in vivo. Importantly, *circLRIG1* was found to sponge *miR-214-3p*, enhancing *LRIG1* expression, and its overexpression also modulated protein levels of E-cadherin, N-cadherin, Vimentin, and LRIG1. Similar effects were observed with *LRIG1* overexpression. Notably, a positive correlation was found between *circLRIG1* and *LRIG1* expression in bladder carcinoma tissues. Additionally, the tumor-suppressing effect of *circLRIG1* was reversed by overexpressing *miR-214-3p* or silencing *LRIG1*. The study concludes that *circLRIG1* suppresses bladder carcinoma progression by enhancing *LRIG1* expression via sponging *miR-214-3p*, providing a potential strategy for early diagnosis and treatment of bladder carcinoma.

## Introduction

Bladder carcinoma is the most prevalent malignant tumor of the urinary system, associated with high morbidity and mortality [1]. As per China’s cancer statistics, approximately 80,500 new cases of bladder carcinoma and 32,900 deaths occurred in 2015 [2]. Bladder cancer is a global health issue with sex differences in incidence and prognosis. Bladder cancer has distinct molecular subtypes with multiple pathogenic pathways depending on whether the disease is non-muscle invasive or muscle invasive [3]. About 70% of bladder carcinoma patients are initially diagnosed with non-muscle-invasive bladder carcinoma, and nearly 30% will develop muscle-invasive carcinoma [4, 5]. Tobacco smoking, exposure to certain chemicals in your diet, environment, or workplace, urinary infections, and certain medications can increase your risk of bladder cancer [6]. Despite advancements in surgical and chemotherapy methods, the 5-year survival rate of bladder carcinoma patients remains disappointingly low [7]. This can be attributed to our limited understanding of the precise molecular mechanisms underlying bladder carcinoma carcinogenesis, which complicates treatment [8]. Recent evidence reveals a plethora of dysregulated transcripts in bladder carcinoma tumorigenesis and progression, including circRNAs [9, 10].

Circular RNAs (circRNAs) are a unique type of non-coding small RNAs, first identified in eukaryotic cells in the 1970s [11]. They are widely expressed in mammalian cells and tissues [12], and are formed by RNA splicing, where the donor site at the 5′ end of an exon is linked to the downstream acceptor site at the 3′ end of an exon [13]. In the past, circRNAs were assumed to be non-functional, resulting from splicing errors. However, with advancements in bioinformatics analysis and high-throughput sequencing, over 30,000 circRNAs have been discovered in various tissues and cells [14]. The potential functions and biological processes of circRNAs remain largely unexplored. Some studies have reported that circRNAs can modulate bladder carcinoma tumorigenesis. For instance, *circRNA FAM114A2* suppresses bladder carcinoma progression by regulating the *miR-762/NP63* axis [15]. *CircMYLK* promotes the proliferation and metastasis of bladder carcinoma through the *miR-34a/CCND3* signaling pathway [16]. *CircRNA_0058063* inhibits bladder carcinoma tumorigenesis by modulating *FOXP4* via *miR-486-3p* sponging [17]. *CircNT5E* contributes to bladder carcinoma tumorigenesis by targeting *miR-502-5p* [18]. *CircRNA_0075828* promotes bladder carcinoma cell growth by increasing the expression of *CREB1* [19]. *circLRIG1*, a newly identified circRNA, has not yet had its potential function and biological processes reported.

MicroRNAs (miRNAs) are a class of small non-coding RNAs approximately 20 nucleotides in length, capable of repressing gene expression by inhibiting translation or promoting mRNA degradation [20]. Numerous studies have reported that abnormal miRNA expression plays a crucial role in many diseases, being intimately associated with metastasis and proliferation processes in bladder carcinoma [21]. *MiR-214-3p*, a cancer-associated microRNA, has been implicated in various cancer progression and tumorigenesis events [22–24]. However, the function of miR-214-3p in cancer is inconsistent, with both promoting and inhibiting effects. For example, Circ_TNFRSF21 promotes cutaneous squamous cell carcinoma metastasis and M2 macrophage polarization via miR-214-3p/CHI3L1 [25]. MiR-214-3p was abnormally elevated in triple negative breast cancer (TNBC), and down-regulation of miR-214-3p could suppress the viability, migration, invasion and EMT of TNBC cells though targeting ST6GAL1, which might be a potential target for future treatment of TNBC [26]. Moreover, miR‑214‑3p promotes the proliferation, migration and invasion of osteosarcoma cells by targeting CADM1 [27]. However, studies investigating the role of *miR-214-3p* in bladder carcinoma are limited. Because only a few references reveal that the expression of miR‑214‑3p in different subtypes of bladder cancer is also different, the expression and function of miR‑214‑3p in bladder cancer, as well as the regulatory mechanism, still need further research. Leucine-rich repeats and immunoglobulin-like domains 1 (*LRIG1*) is a known tumor suppressor gene [28], and it has been proposed as a prognostic indicator for various malignancies, including prostate cancer [29], head-and-neck cancer [30], cutaneous squamous cell carcinoma [31], breast carcinoma [32], and uterine cervical carcinoma [33]. Furthermore, aberrant expression of *LRIG1* has been identified as a significant factor in bladder carcinoma tumorigenesis and progression [34]. The relationships between *circLRIG1*, *miR-214-3p*, and *LRIG1*, however, remain unexplored.

In this study, our results showed that *circLRIG1* was decreased in bladder carcinoma tissues and cell lines, and low expression of *circLRIG1* indicated a poor prognosis for patients with bladder carcinoma. Moreover, overexpression of *circLRIG1* impeded bladder carcinoma cell growth, migration, invasion, and promoted cell apoptosis both in vitro and in vivo. We also demonstrated that *circLRIG1* functioned as a sponge of *miR-214-3p* to modulate the expression of *LRIG1*, and a positive correlation was found between the expression levels of *circLRIG1* and *LRIG1* in bladder carcinoma tissues. These findings shed light on the potential therapeutic strategy for bladder carcinoma patients.

## Materials and methods

### Clinical tissue samples

Bladder carcinoma tissues, along with adjacent non-cancerous specimens, were obtained from 90 patients with bladder carcinoma who underwent surgery at Shanghai Eastern Hepatobiliary Surgery Hospital. All patients had negative histories of exposure to either chemotherapy or radiotherapy before surgical treatment and lacked the co-occurrence of any diagnosed cancers. The cancerous and non-cancerous regions were identified by two professional pathologists. Subsequently, all tissue specimens were stored in liquid nitrogen (Delun, Shanghai, China) within 30 min after resection. Written informed consent was obtained from all participating patients. The clinicopathological characteristics of the 90 patients are presented in Table 1. This research was approved by the Institutional Ethics Committee of Shanghai Eastern Hepatobiliary Surgery Hospital (approval number: [KY-E-2022–3-19]) and was conducted in accordance with the Declaration of Helsinki [35].

**Table 1:** Clinicopathological data of the 90 bladder carcinoma patients

Clinicopathological characteristics	Total	High expression (45)	Low expression (45)	X^2^	P value
Gender					
Male	42	22	20	0.179	0.673
Female	48	23	25		
Age (year)					
≤ 65	40	19	21	0.180	0.671
> 65	50	26	24		
Tumor size					
< 3 cm	34	18	16	0.189	0.664
≥ 3 cm	56	27	29		
Pathology grade					
pTa-pT1	48	31	17	8.750	0.003
pT2-pT4	42	14	28		
Lymph node metastasis					
Positive	51	19	32	7.647	0.006
Negative	39	26	13		
Grade					
Low	44	27	17	4.447	0.035
High	46	18	28		
Muscle invasion					
NMIBC	35	25	10	10.519	0.001
MIBC	55	20	35		

### Cell culture

The normal urothelial cell line SV-HU-1 and bladder carcinoma cell lines (UM-UC-14, SW780, TUCCUP, 5637, J82, UMUC3, BIU87, T24, HT1367, EJ, and RT4) were procured from the ATCC company (ATCC, USA). These cells were cultured in RPMI‑1640 medium (Gibco, USA) supplemented with 10% fetal bovine serum (FBS, Gibco, USA). All cells were maintained at 37 °C in a humidified atmosphere with 5% CO_2_. Complete medium was changed every 2 days, and cells were subcultured when they reached 80% confluence.

### Cell transfection

To construct *circLRIG1* and *LRIG1* overexpression vector (oe-circLRIG1/oe-LRIG1), the full-length of human *circLRIG1* or *LRIG1* were inserted into the pLCDHciR vector (Geenseed Biotech, China), which contained a front and back circular frame, whereas the mock vector with no *circLRIG1* or *LRIG1* sequences were used as a control (vector-circ/vector). To knock down *LRIG1*, siRNAs targeting back splice junction of *LRIG1* (si-*LRIG1*) and a si-NC were synthesized (Geenseed Biotech, China). The siRNA sequence for *LRIG1* was: 5’-GAGCCTAAACCTGATTACAAACA-3’. The mimics control (miR-NC), *miR-214-3p* mimics were procured from the RiboBio company (RiboBio, China). A total of 2 × 10^5^ cells were seeded per well into 6‑well plates and transfected stably with the indicated constructs using Lipofectamine 3000 reagent (Invtrogen, USA) when the cells grew approximately 60–70%. After that, the stably transfected cells were screened with puromycin. Cells after transfection were cultivated at 37 °C with 5% CO_2_ for 24 h for follow-up experiments.

### Cell cytosolic/nuclear fractions assay

In this study, we employed a cell cytosolic/nuclear fractions assay to determine the subcellular localization of *circLRIG1* in bladder carcinoma cell lines (UMUC3 and T24). Briefly, the Cytoplasmic & Nuclear RNA Purification kit (Norgen, Canada) was used to separate nuclear and cytosolic fractions from bladder carcinoma cell lines (UMUC3 and T24), following the manufacturer’s instructions. Total RNA was then extracted from both the cytosolic and nuclear fractions. Subsequently, the subcellular localization of *circLRIG1* in bladder carcinoma cell lines (UMUC3 and T24) was measured using qRT-PCR.

### Actinomycin D and RNase R treatment assay

We employed the Cytoplasmic and Nuclear RNA Purification Kit (Norgen Biotek Corp, Canada) to extract cytoplasmic and nuclear RNAs. Total RNA from bladder carcinoma tissues and cell lines was extracted using TRIzol reagent (Invitrogen, USA). To verify the stability of circRNA and linear mRNA, Actinomycin D (MedChemExpress, China) was added to the medium to halt RNA transcription. Cells were treated with actinomycin D (1 μg/mL) for 0, 8, 16, and 24 h, after which RNA was collected for RT-qPCR analysis. For the RNase R assay, the extracted total RNA from bladder carcinoma cell lines was treated with RNase R (3 U/mg) for 15 min at 37℃, followed by RT-qPCR analysis.

### Quantitative real-time PCR assay

Total RNA from bladder carcinoma tissues and cell lines was collected using Trizol reagent (Invitrogen, USA). The purity and concentration of the RNA were determined by an ultramicrospectrophotometer (NanoDrop-2000c; Thermo Fisher Scientific, MA) The reverse transcription was conducted using a Reverse Transcription kit (Thermo Fisher Scientific, USA). Subsequently, the cDNA was subjected to PCR in a Thermal Cycler Dice Real Time system (Takara, Japan) with SYBR Green PCR master mix (Takara, Japan). The reactions were as follows: at 95 °C for 10 min, 95 °C for 15 s, 60 °C for 60 s, and 70 °C for 1 s, for a total of 40 cycles. The *circLRIG1* and *LRIG1* mRNA levels were normalized by GAPDH. The *miR-214-3p* level was normalized by U6. The experiment was performed in triplicate, and the relative gene expression was calculated using the 2^−ΔΔCt^ method. The primers used for qRT-PCR are listed in Table 2.

**Table 2:** Primers utilized for qPCR

Gene	Forward 5′–3′	Reverse 5′–3′
*circLRIG1*	5’-CCAGAACAGTCGAGGGCAGAA-3’	5’-GGTCCGTGGCCTCTTTAAGTT-3’
*MiR-214-3p*	5’-ACAGCAGGCAGACAGGCAGT-3’	5’-ACTGCCTGTCTGTGCCTGCTGT-3’
*LRIG1*	5’-CTGCATGAGTTGGTCCTGTCC-3’	5’-TGTGGCTGATGGAATTGTGG-3’
U6	5’-CGCTTCCAGCACATATAC-3’	5’-CGCTTCGGCAGCACATATAC-3’
GAPDH	5’-GCGGGGGAGCAAAGGGT-3’	5’-TGGGTGGCAGTGATGGCATGG-3’

### Fluorescence in situ hybridization (FISH)

FISH analysis was performed using a Fluorescent in situ hybridization Kit (Geneseed, Guangzhou, China) according to the manufacturer’s instructions. Cell nuclei were stained with 4,6-diamidino-2-phenylindole (DAPI, Beyotime, China). The images were photographed under the fluorescence microscope (Leica, Wetzlar, Germany) [36].

### Western blot analysis

Bladder carcinoma cell protein extraction was performed according to a previous study [37]. Briefly, proteins from bladder carcinoma cell lines were extracted using RIPA lysis buffer (Sigma-Aldrich, USA) supplemented with a protease inhibitor cocktail (PIC, Roche, USA). Proteins were separated on a 12% SDS-PAGE gel and transferred to a PVDF membrane (Millipore, USA). The membranes were then blocked with 5% BSA at 37 ℃ for 2 h. After washing with PBS, the membranes were incubated with primary antibodies against *LRIG1* (1:2000, CST, USA), N-cadherin (1:2000, CST, USA), E-cadherin (1:1000, Abcam, USA), Vimentin (1:2000, CST, USA), and GAPDH (1:2000, Proteintech, USA) at 4 ℃ for 24 h. Following this, the membrane was incubated with a secondary antibody (1:10,000, Jackson, USA) at 37 ℃ for 2 h. The blots were detected using ECL reagents (Amersham, UK) and evaluated with ImageJ software (National Institutes of Health, USA).

### Wound healing assay

To assess the migratory capability of bladder carcinoma cells, a wound healing assay was conducted. Initially, the cells were seeded into 6-well plates and allowed to reach nearly full confluency. Subsequently, a sterile 1mL pipette tip was used to create a scratch in the cell layer, followed by the addition of serum-free RPMI‑1640 medium. After 48 h, images were captured using an inverted microscope, and the width of the wound was quantified using Image J software.

### CCK-8 assay

The CCK‑8 assay was employed to evaluate cell proliferation. After transfection with the respective constructs for 72 h, cells were harvested and seeded into 96‑well plates at a density of 1 × 10^4 cells/well. Next, cells were incubated for 0, 24, 48, 72, and 96 h. Following each incubation period, 10 µL of CCK‑8 reagent (Beyotime, Shanghai, China) was added to each well and incubated at room temperature for 2 h. The absorbance at a wavelength of 450 nm was then measured using a microplate reader (BioRad Laboratories, USA).

### Colony formation assay

For the colony formation assay, cells were first transfected with the designated constructs for 6 h and then seeded into 6-well plates at a density of 1 × 10^3^ cells/well. After allowing time for colony formation, the cells were fixed with 4% formaldehyde (Thermo Fisher Scientific, USA) and stained with 0.1% crystal violet (Solarbio, Beijing, China). Finally, the number of colonies was counted under a stereomicroscope.

### Flow cytometry analysis

Cells were transfected with specified plasmids for 72 h, following which the cell apoptotic capability was evaluated using the Annexin V-FITC/PI Apoptosis Detection Kit (Thermo Fisher Scientific, USA), in line with the manufacturer's instructions. Subsequently, apoptotic cells were identified using a Beckman Coulter FACS flow cytometer (Beckman Coulter, USA), with the results analyzed by the FlowJo software system (Ashland, USA).

### Transwell assay

Cells transfected with appropriate constructs for 72 h were first starved in serum-free medium. Then, a cell suspension of 0.2 mL of RPMI-1640 medium containing 1 × 10^4^ cells was introduced into the upper chamber (8-μm pore size, Corning, USA) of a Transwell apparatus, either uncoated (for migration assay) or coated with Matrigel (for invasion assay) (BD Biosciences, USA). The lower chamber was filled with RPMI‑1640 medium supplemented with 10% FBS. After a 24-h incubation period, cells were fixed with 4% formaldehyde (Sigma Aldrich, USA) and stained with 1.5% crystal violet (Solarbio, Beijing, China) at 37℃ for 10 min. The The number of migrated and invaded cells penetrating across the membrane was counted under an inverted microscope (Leica, Germany) in five random visual fields, and all experiments were repeated in triplicate.

### Dual-luciferase reporter assay

Luciferase reporter vectors carrying either the wild-type (WT) or mutant (Mut) sequences of *circLRIG1* or *LRIG1* were inserted into a psiCHECK-2 vector (Promega Corporation), downstream of the Renilla luciferase gene. The vector also contained the Firefly luciferase gene. A total of 4 × 10^5^ UMUC3 and T24 cells were seeded per well into 6-well plates and co-transfected with either mimic control or *miR-214-3p* mimics and plasmid vector using Lipofectamine 3000 reagent (Invtrogen, USA). After 72 h of transfection, cells were harvested, and luciferase activities were measured using the Luciferase Reporter Assay System (Promega, USA). Each experiment was performed in triplicate.

### RNA pull-down assay

The interaction between *circLRIG1*, *miR-214-3p*, and *LRIG1* in bladder carcinoma cells was confirmed using an RNA pull-down assay. Biotin-labeled miR-214-3p mimics (miR-214-3p probe) and negative control mimics (NC probe) were purchased from GenePharma (China). Briefly, cells were transfected with either *miR-214-3p* probe or NC-probe using Lipofectamine 3000 (Invtrogen, USA), according to the manufacturer's instructions. After a 48-h post-transfection period, cells were harvested, and samples were incubated with magnetic beads (Life Technologies, USA). After three washes with PBS, the mRNA levels of *circLRIG1* and *LRIG1* were quantified by qRT-PCR [38].

### RNA immunoprecipitation (RIP) assay

To confirm the relationship between *circLRIG1*, *miR-214-3p*, and *LRIG1* in bladder carcinoma cells, we used the RNA immunoprecipitation assay. Briefly, cells were treated with RIP lysis buffer, and the anti-Ago2 antibody (1:2000; Abcam; USA) or normal IgG antibody (1:2000; Abcam; USA) was conjugated to magnetic beads and incubated with the cell extracts at 4°C for 24 h. The magnetic beads were then harvested and incubated with proteinase K. Finally, we assessed the enrichment of *circLRIG1*, *miR-214-3p*, and *LRIG1* in the immunoprecipitated RNA by qRT-PCR analysis.

### Xenograft tumor model

Nude mice were obtained from Shanghai SLAC Laboratory Animal company (Shanghai, China). Our animal research was approved by the Ethics Review Committee of Shanghai Eastern Hepatobiliary Surgery Hospital (IACUC permit number: SW2022-102109). We subcutaneously injected UMUC3 bladder carcinoma cells (1 × 10^6^/mice) transfected with OE-vector or OE-*circLRIG1* plasmids suspended in PBS into the mice. We calculated the tumor volume weekly, and after 28 days, the mice were sacrificed, and the tumor tissue was weighed.

### Hematoxylin and eosin staining

We sliced the fixed sections of lung tissues in the mid-sagittal plane, paraffin-embedded, and cut into sections. Next, we subjected the sections to deparaffinization and rehydration, stained them with hematoxylin and eosin (HE) for morphological analysis, and visualized them under an Olympus BX41 microscope (Tokyo, Japan). We calculated the number of metastatic nodules in the lungs.

### Statistical analyses

All experimental data were analyzed using Prism 7.0 software. The data were presented as mean ± SD. We used Student’s t-test to analyze the data between two groups and ANOVA to analyze data between multiple groups. We used the Kaplan–Meier (K-M) plot to analyze the survival curves. The log-rank test was used to analyze the difference between K-M curves. We analyzed the correlation between the expression of *circLRIG1* and *LRIG1* in bladder carcinoma patient tissues using the Pearson χ2 test. A P value of less than 0.05 was considered to be statistically significant.

## Results

### *CircLRIG1* is downregulated in bladder carcinoma tissues and cell lines

**Fig. 1:**
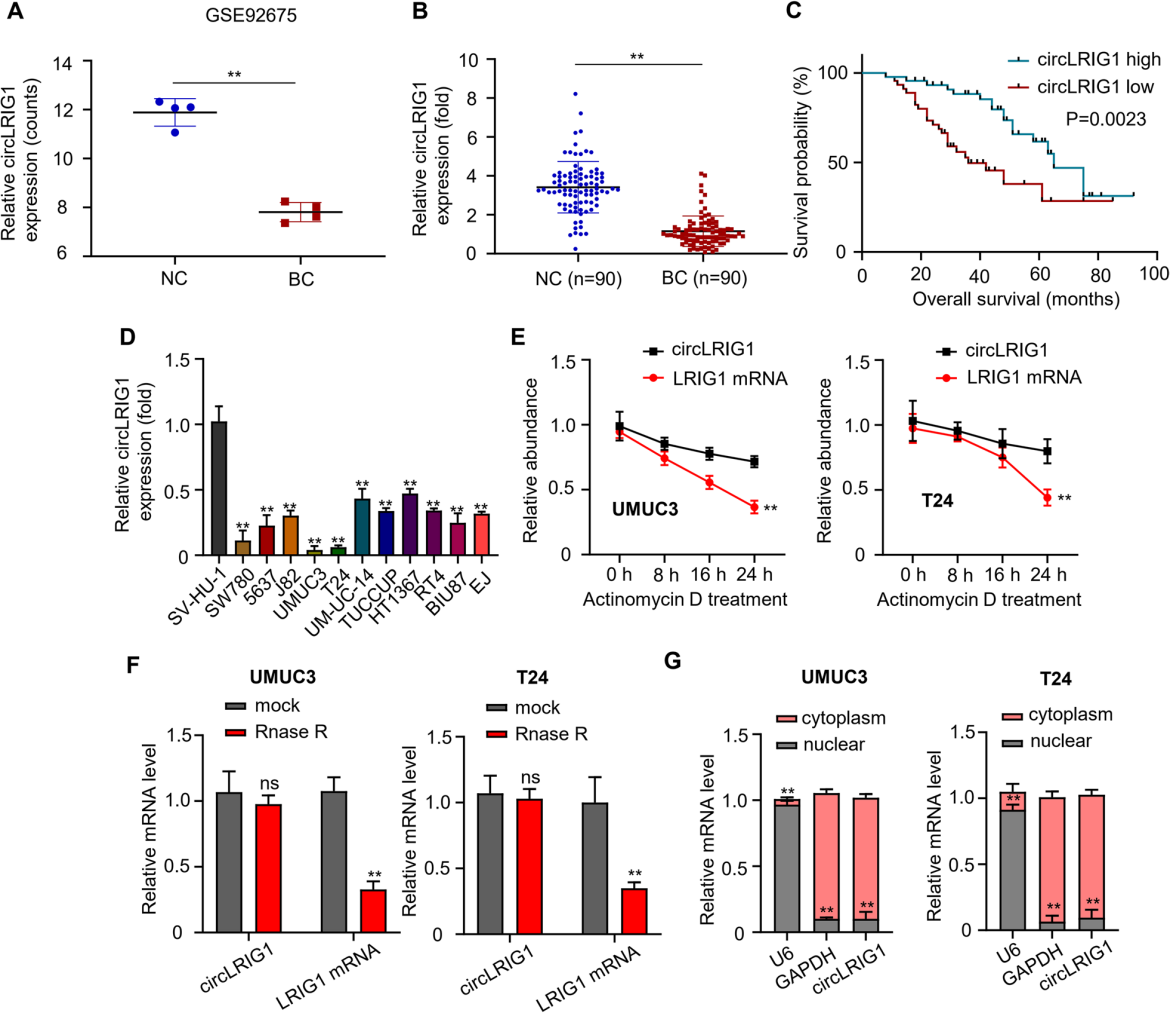
Expression of *circLRIG1* in bladder carcinoma. **A** Analysis of *circLRIG1* expression in bladder carcinoma tissues and adjacent noncancerous specimens using the GSE92675 database. **B** qRT-PCR analysis of *circLRIG1* mRNA levels in 90 pairs of bladder carcinoma tissues and adjacent noncancerous specimens. **C** K-M survival analysis of bladder carcinoma patients with low or high expression of *circLRIG1*. **D** qRT-PCR analysis of *circLRIG1* mRNA levels in normal urothelial cell line SV-HU-1 and 11 bladder carcinoma cell lines (UM-UC-14, SW780, TUCCUP, 5637, J82, UMUC3, BIU87, T24, HT1367, EJ and RT4). **E** qRT-PCR analysis of *circLRIG1* and *LRIG1* mRNA levels in bladder carcinoma cell lines (UMUC3 and T24) treated with Actinomycin D. **F** qRT-PCR analysis of *circLRIG1* and *LRIG1* mRNA levels in bladder carcinoma cell lines (UMUC3 and T24) with or without RNase R treatment. **G** Subcellular localization of *circLRIG1* in bladder carcinoma cell lines (UMUC3 and T24) confirmed by cytosolic/nuclear fractionation assay. Experiments were performed in triplicate. “**” indicates *P* < 0.01

To identify bladder carcinoma-associated circRNAs, we analyzed RNA sequencing datasets from the GSE92675 database and found that the mRNA level of *circLRIG1* was decreased in bladder carcinoma tissues compared to adjacent noncancerous tissues (Fig. 1A). Subsequently, we validated the expression of *circLRIG1* in 90 pairs of bladder carcinoma tissues and adjacent noncancerous specimens using qRT-PCR. Our results showed that *circLRIG1* expression was significantly reduced in bladder carcinoma tissues (Fig. 1B). K-M analysis indicated that lower *circLRIG1* expression was associated with poorer overall survival in bladder carcinoma patients (Fig. 1C). We also observed a significant decrease in *circLRIG1* expression in bladder carcinoma cell lines compared to normal urothelial cells (SV-HU-1) and that *circLRIG1* was markedly reduced in UMUC3 and T24 cells compared to other bladder carcinoma cell lines (Fig. 1D). We further confirmed the stability of *circLRIG1* compared to linear *LRIG1* using Actinomycin D and RNase R Treatment assays, which showed that *circLRIG1* was more stable than linear *LRIG1* (Fig. 1E) and was resistant to RNase R digestion (Fig. 1F). Furthermore, we determined the subcellular localization of *circLRIG1* in UMUC3 and T24 cells using the cell cytosolic/nuclear fractions assay, which revealed that *circLRIG1* was mainly located in the cytoplasm of bladder carcinoma cells (Fig. 1G). Based on the median value of *circLRIG1* expression, we divided bladder carcinoma samples into low and high *circLRIG1* groups and found that *circLRIG1* expression was significantly associated with Pathology grade, Lymph node metastasis, and Muscle invasion in bladder carcinoma patients (Table 1). These results suggest that *circLRIG1* may play a potential role in the progression of bladder carcinoma.

### Upregulation of *circLRIG1* suppresses cell proliferation in bladder carcinoma

**Fig. 2:**
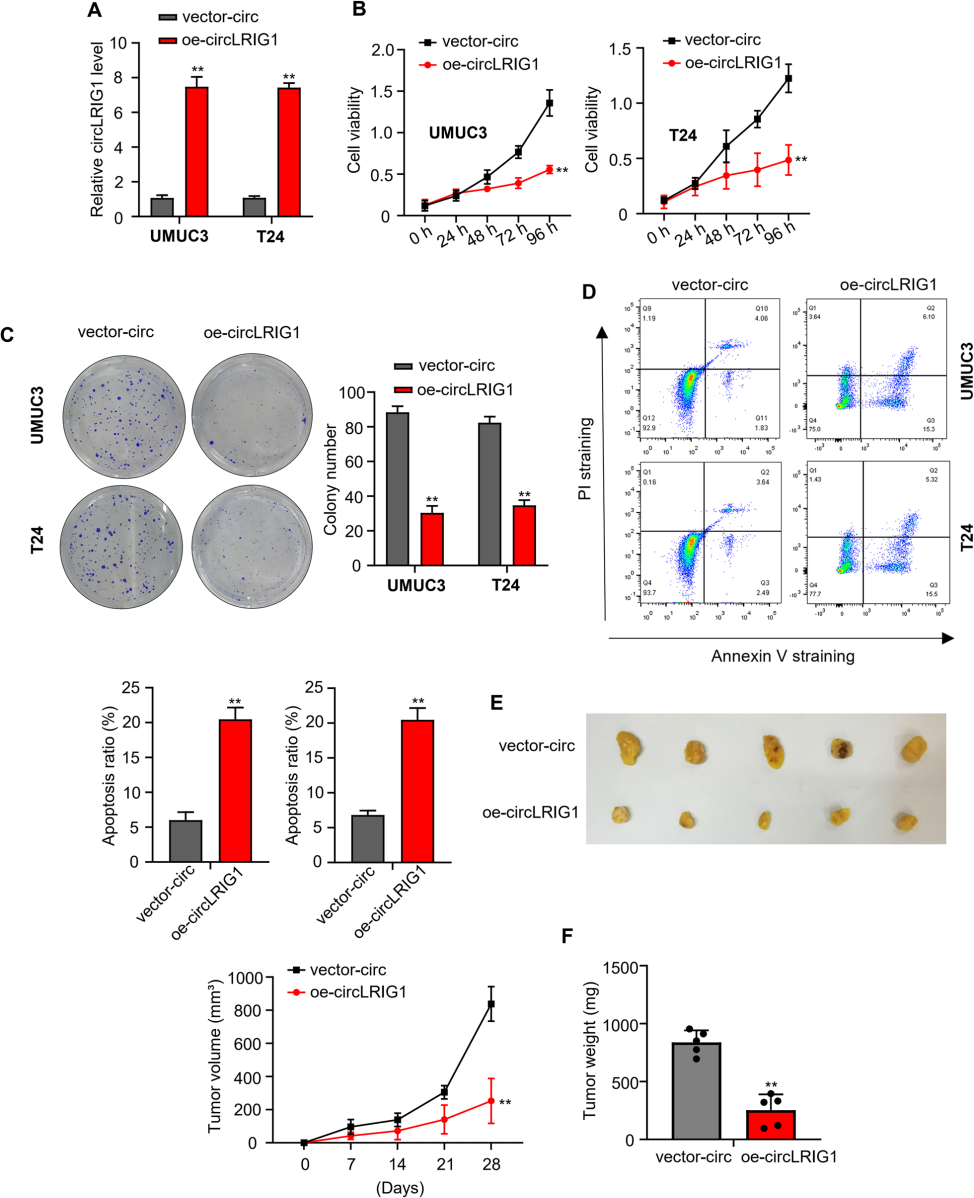
Upregulation of *circLRIG1* suppresses bladder carcinoma cell proliferation. **A** qRT-PCR analysis of *circLRIG1* mRNA levels in bladder carcinoma cell lines (UMUC3 and T24) transfected with OE-vector or OE-*circLRIG1* plasmids. **B** Cell viability in (**A**) was determined by CCK-8 assay. **C** Colony formation assay to determine the colony number of cells in (**A**). **D** Flow cytometry analysis to determine apoptosis rate of cells in (**A**). **E** Tumor volume was measured at 0, 7, 14, 21, and 28 days. **F** Tumor weight was measured at 28 days of the xenograft experiment. Experiments were performed in triplicate. “**” indicates *P* < 0.01

In vitro assays were performed to elevate the mRNA level of *circLRIG1* in UMUC3 and T24 cells through transfection with OE-*circLRIG1* plasmids (Fig. 2A). To further explore the role of *circLRIG1* in bladder carcinoma carcinogenesis and progression, CCK-8 and colony formation assays were used to determine cell proliferation ability. Our data showed that *circLRIG1* overexpression inhibited UMUC3 and T24 cell viability (Fig. 2B) and reduced cell colony number (Fig. 2C). Next, Flow cytometry analysis was utilized to determine cell apoptosis abilities. Our data revealed that cell apoptosis ability was significantly enhanced by transfection with OE-*circLRIG1* plasmids in UMUC3 and T24 cells (Fig. 2D).

In vivo study, nude mice were subcutaneously injected with bladder carcinoma UMUC3 cells (cells transfected with OE-vector or OE-*circLRIG1* plasmids). Tumor volumes were calculated 7 days after injection. The mice were sacrificed 28 days after cell injection, and the volume and weight of isolated tumors were calculated. Our data showed that *circLRIG1* overexpression was associated with lower tumor growth rates (Fig. 2E) and reduced tumor weight (Fig. 2F), suggesting that *circLRIG1* might work as a tumor suppressor gene in the proliferation of bladder carcinoma.

### Upregulation of *circLRIG1* suppresses cell metastasis in bladder carcinoma

**Fig. 3:**
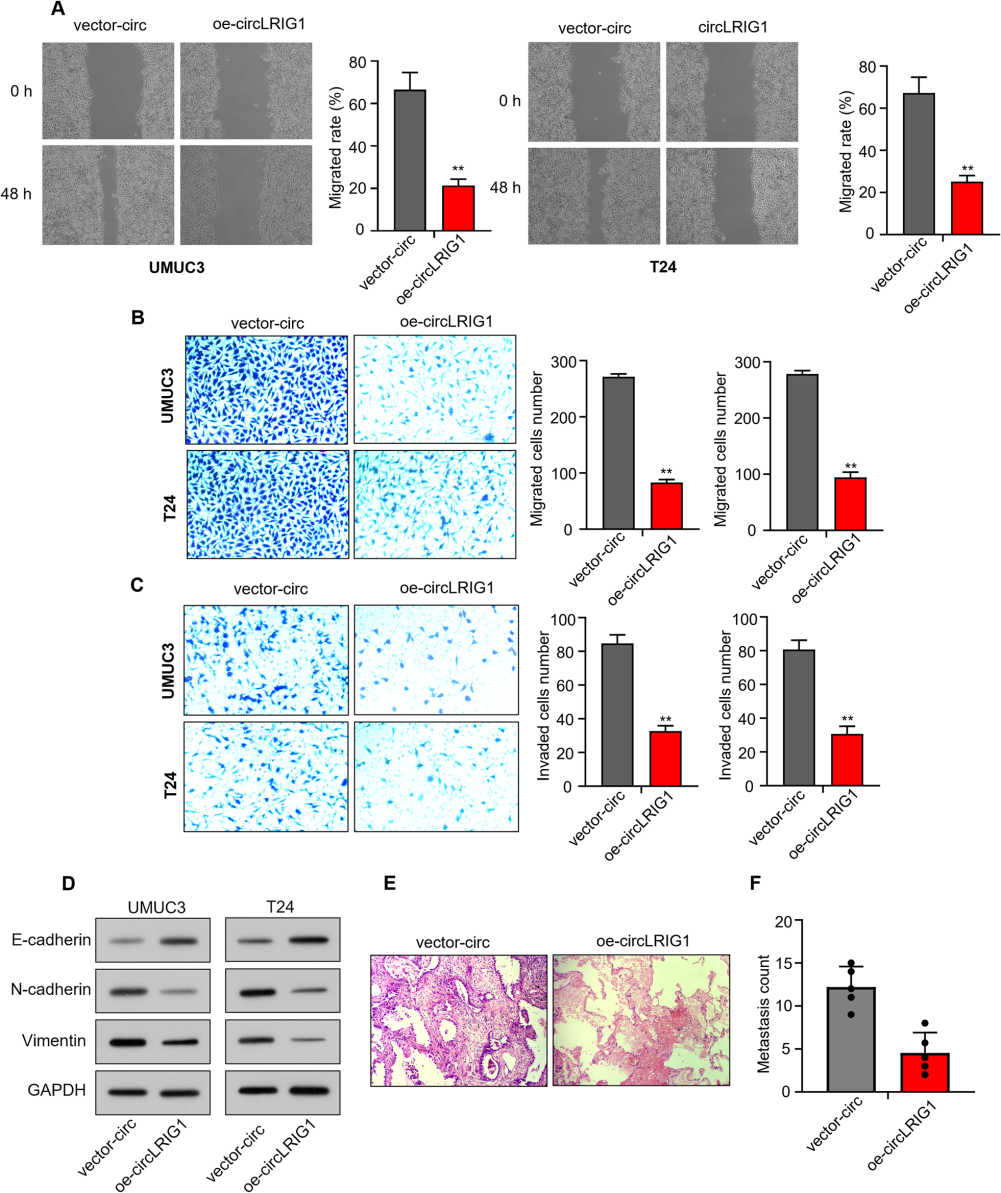
Upregulation of *circLRIG1* suppresses cell metastasis in bladder carcinoma. **A** The number of migrated cells in bladder carcinoma cell lines (UMUC3 and T24) after transfection with OE-vector or OE-*circLRIG1* plasmids was determined using the wound healing assay. **B** The cell migration capability in (**A**) was determined using the Transwell assay (uncoated with Matrigel). **C** The cell invasion capability in (**A**) was determined using the Transwell assay (coated with Matrigel). **D** The protein levels of E-cadherin, N-cadherin, and Vimentin in bladder carcinoma cell lines (UMUC3 and T24) after transfection with OE-vector or OE-*circLRIG1* plasmids were determined using the Western blot assay. **E** The number of lung metastatic nodules was determined using H&E staining. **F** The number of lung metastatic nodules was counted.The experiment was performed in triplicate. “**” indicates *P* < 0.01

We performed wound healing and Transwell assays to measure the metastatic capability of bladder carcinoma cells. The wound healing assay showed that *circLRIG1* overexpression could significantly reduce the number of migrated cells in bladder carcinoma cell lines (UMUC3 and T24) (Fig. 3A). The Transwell assay demonstrated that *circLRIG1* overexpression could inhibit the migration and invasion capability of UMUC3 and T24 cells (Fig. 3B, C). Furthermore, we detected the protein levels of E-cadherin, N-cadherin, and Vimentin in bladder carcinoma cell lines (UMUC3 and T24) after transfection with OE-*circLRIG1* plasmids by utilizing Western blot analysis. Results showed that *circLRIG1* overexpression could elevate the protein level of E-cadherin while reducing the protein levels of N-cadherin and Vimentin in bladder carcinoma cells (Fig. 3D).

In an in vivo study to assess the role of *circLRIG1* in bladder carcinoma cell metastasis, we injected bladder carcinoma UMUC3 cells infected with OE-vector or OE-*circLRIG1* plasmids into NOD/SCID mice. The Hematoxylin and eosin staining assay revealed that lung metastasis was dramatically inhibited by inoculation with UMUC3-OE-*circLRIG1* (Fig. 3E). Additionally, the number of lung metastatic nodules was significantly reduced in mice injected with UMUC3-OE-*circLRIG1* (Fig. 3F). These data suggest that *circLRIG1* functions as a tumor suppressor gene in the metastasis of bladder carcinoma.

### Upregulation of *LRIG1* suppresses progression of bladder carcinoma

**Fig. 4:**
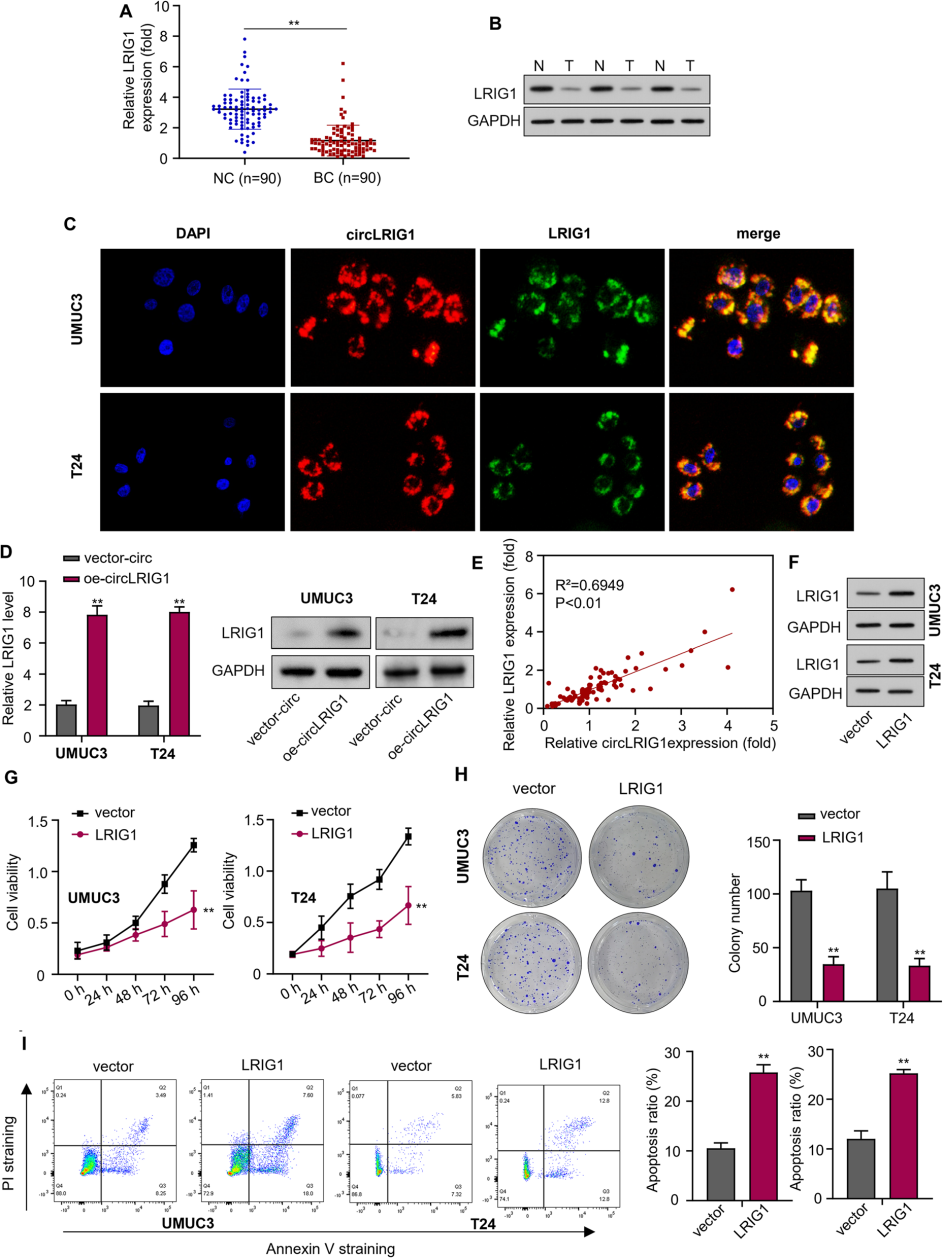
Upregulation of *LRIG1* suppresses the progression of bladder carcinoma. **A** The mRNA level of *LRIG1* in 90 pairs of bladder carcinoma tissues and adjacent noncancerous specimens was determined using the qRT-PCR assay. **B** The protein level of *LRIG1* in 3 pairs of bladder carcinoma tissues and adjacent noncancerous specimens was determined using the Western blot assay. **C** The co-localization of *circLRIG1* and *LRIG1* was observed in UMUC3 and T24 cells (magnification, × 200, Scale bar, 50 μm) by FISH assay. **D** The mRNA and protein levels of *LRIG1* in bladder carcinoma cell lines (UMUC3 and T24) after transfection with vector-circ or oe-*circLRIG1* plasmids were determined using the qRT-PCR and Western blot assay. **E** The correlation between the expression of *circLRIG1* and *LRIG1* in bladder carcinoma tissues was analyzed using Pearson’s correlation coefficient. **F** The protein levels of *LRIG1* in bladder carcinoma cell lines (UMUC3 and T24) after transfection with OE-vector or OE-*LRIG1* plasmids were determined using the Western blot assay. **G** The cell viability was determined using the CCK-8 assay. **H** The number of cell colonies was determined using the colony formation assay. **I** The number of apoptotic cells was determined using the flow cytometry analysis. The experiment was performed in triplicate. “**” indicates *P* < 0.01

**Fig. 5:**
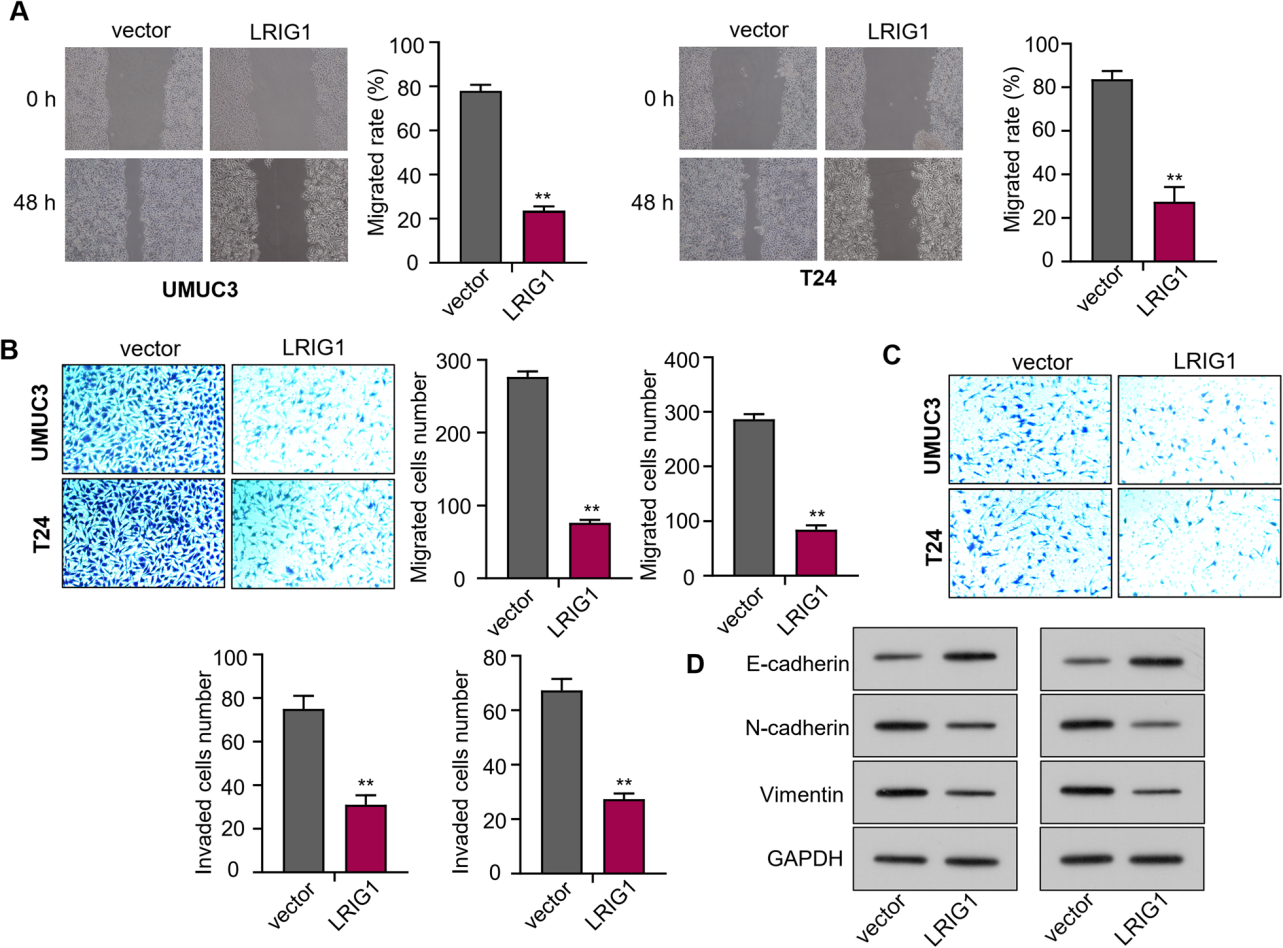
Upregulation of *LRIG1* suppresses cell metastasis in bladder carcinoma. **A** The number of migrated cells was determined using the wound healing assay. **B** The cell migration capability was determined using the Transwell assay (uncoated with Matrigel). **C** The cell invasion capability was determined using the Transwell assay (coated with Matrigel). **D** The protein levels of E-cadherin, N-cadherin, and Vimentin in bladder carcinoma cell lines (UMUC3 and T24) after transfection with OE-vector or OE-*LRIG1* plasmids were determined using the Western blot assay. The experiment was performed in triplicate. “**” indicates *P* < 0.01

We first detected the mRNA level of *LRIG1* in bladder carcinoma tissues and adjacent noncancerous specimens using qRT-PCR assay. The results showed that *LRIG1* expression was significantly lower in bladder carcinoma tissues compared to adjacent noncancerous specimens (Fig. 4A). We also examined the protein expression of *LRIG1* in three pairs of bladder carcinoma tissues and adjacent noncancerous specimens using Western blot analysis, and the data suggested that the protein expression of *LRIG1* was decreased in bladder carcinoma tissues (Fig. 4B). Before studying the relationship between *circLRIG1* and *LRIG1*, we investigated the subcellular colocalization of *circLRIG1* and *LRIG1* in UMUC3 and T24 cells by FISH assay. We found that *circLRIG1* was co-localized with *LRIG1* in the cytoplasm (Fig. 4C). We then used qRT-PCR and Western blot analysis to assess the expression levels of *LRIG1* in BCa cell lines. The results indicated that *LRIG1* were upregulated when *circLRIG1* was overexpressed in UMUC3 and T24 cells (Fig. 4D). Moreover, we found a positive correlation between the expression levels of *circLRIG1* and *LRIG1* in bladder carcinoma tissues (Fig. 4E). To investigate the role of *LRIG1* in bladder carcinoma, we overexpressed *LRIG1* in UMUC3 and T24 cells by transfecting them with OE-*LRIG1* plasmids, and the Western blot analysis confirmed the elevated protein level of *LRIG1* (Fig. 4F). Our data revealed that overexpression of *LRIG1* inhibited cell viability (Fig. 4G) and colony formation (Fig. 4H) in UMUC3 and T24 cells. Furthermore, flow cytometry analysis showed that *LRIG1* overexpression significantly increased cell apoptosis (Fig. 4I). To explore the effect of *LRIG1* on bladder carcinoma cell metastasis capability, we performed wound healing and Transwell assays. The results showed that overexpression of *LRIG1* significantly inhibited the migration and invasion of UMUC3 and T24 cells (Fig. 5A–C). Additionally, Western blot analysis revealed that *LRIG1* overexpression increased the protein level of E-cadherin but decreased the protein level of N-cadherin and Vimentin in bladder carcinoma cells (Fig. 5D). These results suggest that *LRIG1* may function as a tumor suppressor gene in the progression of bladder carcinoma.

### *CircLRIG1* regulates *LRIG1* expression by sponging *miR-214-3p*

**Fig. 6:**
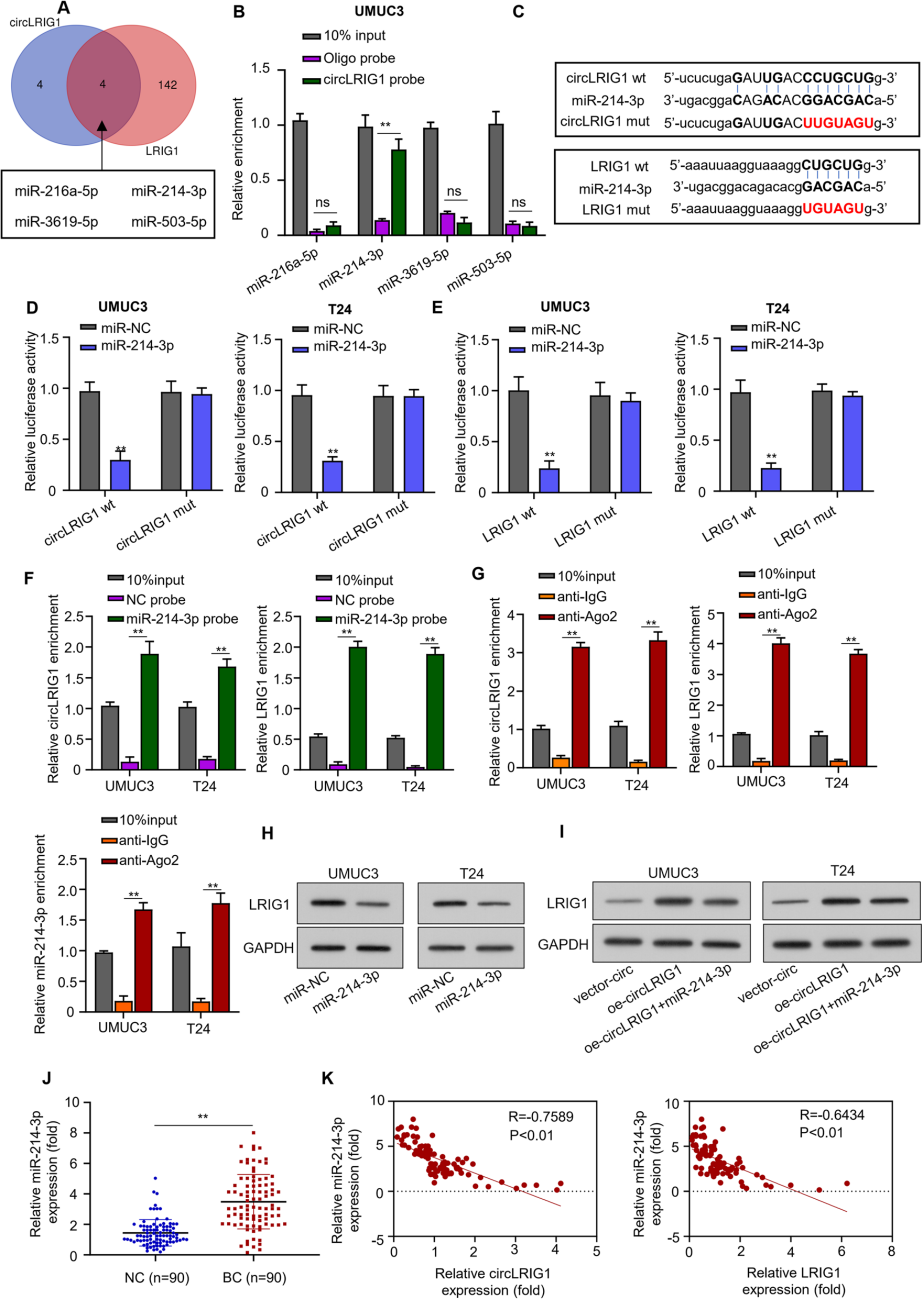
*circLRIG1* regulates *LRIG1* expression by sponging *miR-214-3p*. **A** Venn diagram showing the overlap of miRNAs targeted by *circLRIG1* and *LRIG1* in bladder carcinoma cells. **B** RNA pull-down assay using *circLRIG1* and Oligo probes was performed in UMUC3 cells. **C** Potential targets of wild-type and mutant *circLRIG1* or *LRIG1* on *miR-214-3p*. **D** Relationship between *circLRIG1* and *miR-214-3p* was determined using the luciferase reporter assay. **E** Relationship between *miR-214-3p* and *LRIG1* was determined using the luciferase reporter assay. **F** Relationship between *circLRIG1* and *miR-214-3p* or *miR-214-3p* and *LRIG1* was determined using the RNA pull-down assay. **G** Relationship between *circLRIG1* and *miR-214-3p* or *miR-214-3p* and *LRIG1* was determined using the RNA immunoprecipitation (RIP) assay. **H** Protein level of *LRIG1* in bladder carcinoma cell lines (UMUC3 and T24) was determined by Western blot assay after transfection with indicated constructs (*miR-214-3p* mimics, mimics control). **I** Protein level of *LRIG1* in bladder carcinoma cell lines (UMUC3 and T24) was determined by Western blot assay after transfection with indicated constructs (OE-vector, OE-*circLRIG1*, OE-*circLRIG1* + *miR-214-3p* mimics). **J** QRT-PCR analysis of *miR-214-3p* mRNA levels in 90 pairs of bladder carcinoma tissues and adjacent noncancerous specimens. **K** The correlation between the expression of *miR-214-3p and circLRIG1* as well as *LRIG1* in bladder carcinoma tissues was analyzed using Pearson’s correlation coefficient. The experiment was performed in triplicate. “**” represents *P* < 0.01

To identify the miRNAs targeted by *circLRIG1* and *LRIG1*, we used the online tool StarBase 2.0 and obtained four miRNAs that overlapped: miR-216a-5p, *miR-214-3p*, miR-3619-5p, and miR-503-5p (Fig. 6A). Using a *circLRIG1* probe, we performed an RNA pull-down assay in UMUC3 cells and found that *circLRIG1* and *miR-214-3p* were significantly enriched (Fig. 6B). Further analysis using StarBase 2.0 predicted *miR-214-3p* as the potential target of *circLRIG1* (Fig. 6C). We confirmed this interaction using a luciferase reporter assay and found that co-transfection of the wild-type *circLRIG1* with *miR-214-3p* mimics in bladder carcinoma cell lines (UMUC3 and T24) reduced the luciferase reporter activity, whereas the mutant *circLRIG1* did not (Fig. 6D).

Additionally, we found that *LRIG1* was a potential target gene of *miR-214-3p* using StarBase 2.0 (Fig. 6C). Using a luciferase reporter assay, we confirmed this interaction and found that co-transfection of the wild-type *LRIG1* with *miR-214-3p* mimics in bladder carcinoma cell lines (UMUC3 and T24) reduced the luciferase reporter activity, but not the mutant *LRIG1* (Fig. 6E). In a biotinylated *miR-214-3p* probe RNA pull-down assay, we found that *circLRIG1* and *LRIG1* were detected in the *miR-214-3p*-probe compared to the NC-probe (Fig. 6F). The RNA immunoprecipitation (RIP) assay further demonstrated that the Ago2 antibody precipitated the Ago2 protein from the cell lysates, and the expression of *circLRIG1*, *miR-214-3p*, and *LRIG1* increased in the Ago2 pellet (Fig. 6G).

We next transfected bladder carcinoma cell lines (UMUC3 and T24) with *miR-214-3p* mimics and found that the protein expression of *LRIG1* was reduced (Fig. 6H). Conversely, transfection with OE-*circLRIG1* elevated the protein levels of *LRIG1* expression, but co-transfection with *miR-214-3p* mimics reversed this effect (Fig. 6I). Subsequently, we validated the expression of *miR-214-3p* in 90 pairs of bladder carcinoma tissues and adjacent noncancerous specimens using qRT-PCR. Our results showed that *miR-214-3p* expression was significantly induced in bladder carcinoma tissues (Fig. 6J). Moreover, we found the negative correlations between the expression levels of *miR-214-3p* and *circLRIG1* as well as *LRIG1* in bladder carcinoma tissues (Fig. 6K). These findings suggest that *circLRIG1* regulates the expression of *LRIG1* by sponging *miR-214-3p*.

### *CircLRIG1* suppresses the progression of bladder carcinoma by modulating the *miR-214-3p*/*LRIG1* axis

**Fig. 7:**
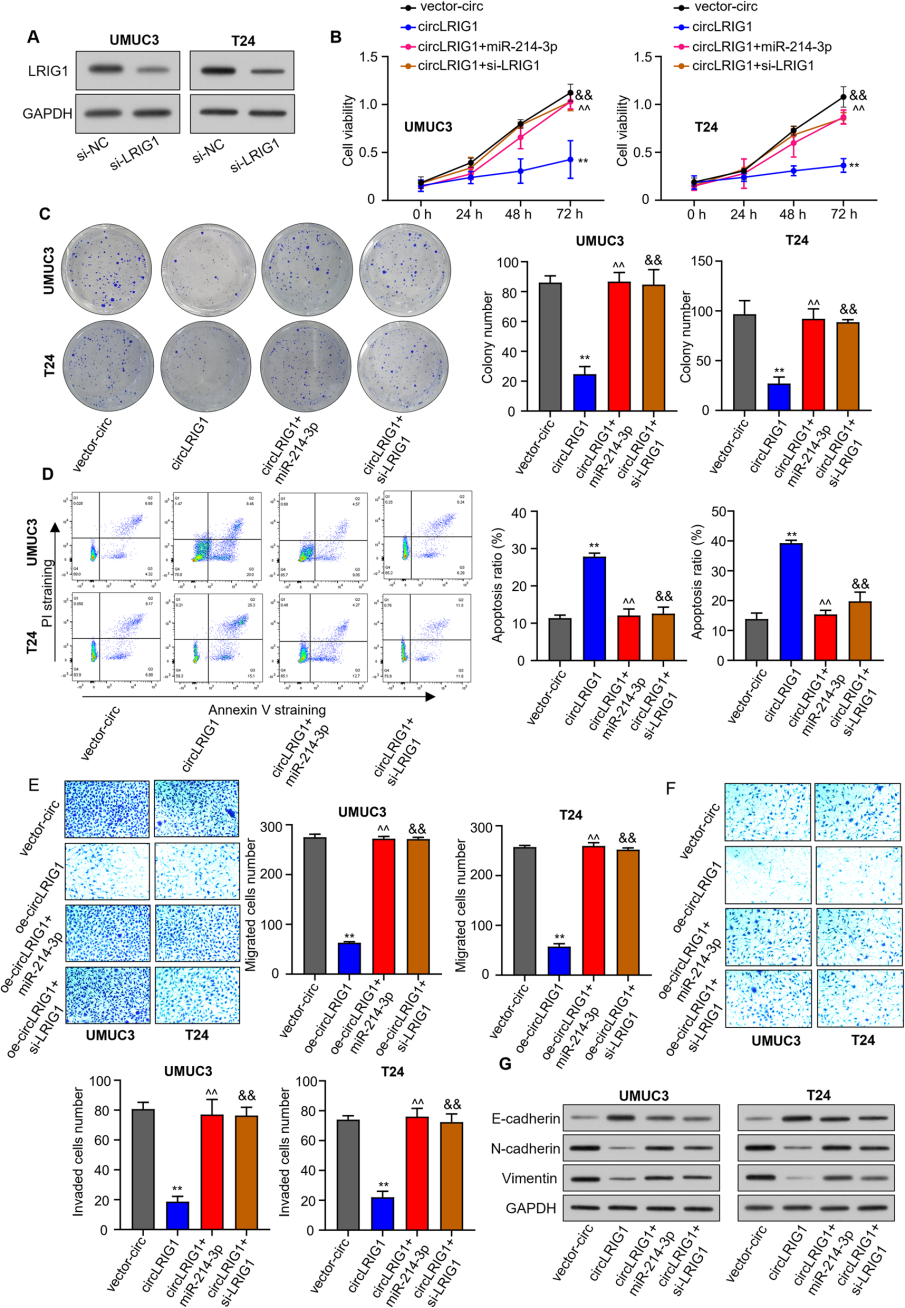
*circLRIG1* suppresses bladder carcinoma progression by modulating the *miR-214-3p*/*LRIG1* axis. **A** Protein level of *LRIG1* in bladder carcinoma cell lines (UMUC3 and T24) was determined by Western blot assay after transfection with si-NC or si-*LRIG1*. **B** Cell viability of bladder carcinoma cell lines (UMUC3 and T24) was determined by CCK-8 assay after transfection with indicated constructs (OE-vector, OE-*circLRIG1*, OE-*circLRIG1* + *miR-214-3p* mimics, OE-*circLRIG1* + si-*LRIG1*). **C** Colony formation assay was used to determine the colony number of cells in (**B**). **D** Flow cytometry analysis was used to determine the apoptosis number of cells in (**B**). **E** Transwell assay (uncoated with Matrigel) was used to determine the cell migration capability in (**B**). **F** Transwell assay (coated with Matrigel) was used to determine the cell invasion capability in (**B**). **G** Protein level of E-cadherin, N-cadherin, and Vimentin was determined by Western blot assay in (**B**). The experiment was performed in triplicate. ^**^P < 0.01, ^^^^*P* < 0.01, ^&&^*P* < 0.01

To investigate whether *circLRIG1* is involved in the progression of bladder carcinoma by regulating the *miR-214-3p*/*LRIG1* axis, we first silenced *LRIG1* in bladder carcinoma cell lines (UMUC3 and T24) by transfecting them with si-*LRIG1*. Our data revealed that the protein levels of *LRIG1* expression were reduced in UMUC3 and T24 cells after transfection with si-*LRIG1* (Fig. 7A). In vitro studies showed that *circLRIG1* overexpression inhibited cell proliferation, colony formation, migration, invasion and promoted cell apoptosis. However, these effects were abrogated by co-transfection with *miR-214-3p* mimics or si-*LRIG1* (Fig. 7B–F). Finally, we used Western blot analysis to detect the protein levels of E-cadherin, N-cadherin and Vimentin in bladder carcinoma cell lines (UMUC3 and T24). The results showed that *circLRIG1* overexpression increased the protein level of E-cadherin while reducing the protein level of N-cadherin and Vimentin in bladder carcinoma cells. However, these effects were reversed by co-transfection with *miR-214-3p* mimics or si-*LRIG1* (Fig. 7G). These results demonstrate that *circLRIG1* can suppress the progression of bladder carcinoma by modulating the *miR-214-3p*/*LRIG1* axis.

## Discussion

With the development of bioinformatics analysis and high-throughput sequencing, numerous circRNAs have been identified in various genomes, most of which are highly stable and widely expressed in different tissues and cells [39]. Increasing evidence has shown that circRNAs can modulate gene expression and play a crucial role in the tumorigenesis and progression of various malignant tumors. For instance, circPTK2 facilitates cell proliferation in gastric cancer by sponging miR-139-3p [40]. CircPDHX accelerates cell proliferation and metastasis in prostate carcinoma by targeting miR-378a-3p [41]. CirRNA_0014717 suppresses the progression of hepatocellular cancer by modulating the miR-668-3p/BTG2 signaling pathway [42]. However, the precise molecular mechanism of circRNAs in bladder carcinoma tumorigenesis and progression remains to be further studied. In our study, we identified a new bladder carcinoma-associated circRNA, *circLRIG1*, which was significantly downregulated in bladder carcinoma tissues and cells, and mainly located in the cytoplasm of bladder carcinoma cells. The expression level of *circLRIG1* was negatively associated with pathology grade, lymph node metastasis, and muscle invasion in bladder carcinoma patients. K-M analysis showed that lower *circLRIG1* expression predicted poor prognosis in bladder carcinoma patients. We further confirmed the stability of *circLRIG1* and linear *LRIG1* using Actinomycin D and RNase R Treatment assays. Additionally, our data demonstrated that *circLRIG1* functions as a tumor suppressor gene, impairing the proliferation and metastasis of bladder carcinoma both in vivo and in vitro. These results suggest that *circLRIG1* may be involved in the tumorigenesis and progression of bladder carcinoma.

The research on the posttranscriptional regulation of circRNAs has primarily focused on the function of miRNA sponges [43]. CircRNAs contain many miRNA response elements and miRNA-binding sites that can function as miRNA sponges and have been shown to be involved in tumorigenesis in various malignant tumors, including bladder carcinoma. For example, circ_0004463 functions as a ceRNA for miR-380-3p and is involved in bladder carcinoma cell apoptosis by regulating mitochondrial respiration process [44]. CircDOCK1 promotes bladder carcinoma tumorigenesis by sponging miR-132-3p [45]. CircCEP128 has also been found to act as a miR-145-5p sponge and accelerate bladder carcinoma tumorigenesis [46]. However, there have been no reports on the miRNA sponging function of *circLRIG1*. In our study, we used bioinformatics analysis to identify a binding sequence for *miR-214-3p* in *circLRIG1*. We further confirmed the interaction between *circLRIG1* and *miR-214-3p* in bladder carcinoma through the use of the Luciferase reporter assay, RNA immunoprecipitation (RIP) assay, RNA pull-down assay, and Western blot analysis. These results suggest that *circLRIG1* exerts its biological function through sponging *miR-214-3p*.

As is well-known, miRNAs suppress gene expression through translational repression or mRNA degradation [47]. Herein, through bioinformatics prediction, we found that *miR-214-3p* can bind to the 3’-UTR of *LRIG1*. Using the Luciferase reporter assay, RIP assay, RNA pull-down assay, and Western blot analysis, we confirmed that *miR-214-3p* can directly target the *LRIG1* gene. *LRIG1* is located on chromosome 3p14.3, and the *LRIG1* protein is a transmembrane protein composed of an extracellular region consisting of three immunoglobulin-like domains and fifteen leucine-rich repeats. Previous studies have shown that *LRIG1* is associated with the progression of many malignant tumors, including bladder carcinoma [48–50]. In our study, we confirmed that *LRIG1* is diminished in bladder carcinoma tissues and cell lines. We also demonstrated that *LRIG1* can serve as a tumor suppressor gene and impair the proliferation and metastasis of bladder carcinoma in vitro. These findings reveal that *LRIG1* serves as a tumor suppressor gene in bladder carcinoma tumorigenesis and progression. Furthermore, we found that overexpression of *circLRIG1* can impair cell growth, migration, invasion, and promote cell apoptosis in bladder carcinoma cells, but overexpression of *miR-214-3p* or *LRIG1* silencing can reverse these effects. These results elucidate that *circLRIG1* suppresses the progression of bladder carcinoma through modulating the *miR-214-3p*/*LRIG1* signaling pathway.

In summary, our study reveals for the first time that *circLRIG1* is diminished in bladder carcinoma tissue and cell lines. *circLRIG1* can elevate the expression of *LRIG1* by sponging *miR-214-3p* to repress the tumorigenesis and progression of bladder carcinoma. Our findings provide a new strategy for early diagnosis and therapeutic intervention for bladder carcinoma.

## Data Availability

The datasets used and/or analyzed during the current study are available from the corresponding author on reasonable request.
